# Expression of TRPS1 in Metastatic Tumors of the Skin: An Immunohistochemical Study of 72 Cases

**DOI:** 10.3390/dermatopathology11040031

**Published:** 2024-10-23

**Authors:** Kassiani Boulogeorgou, Christos Topalidis, Triantafyllia Koletsa, Georgia Karayannopoulou, Jean Kanitakis

**Affiliations:** 1 Laboratory of Pathology, AHEPA University Hospital, Aristotle University of Thessaloniki, 541 24 Thessaloniki, Greece; siliaboulog@gmail.com (K.B.); xtopalidis94@gmail.com (C.T.); tkoletsa@auth.gr (T.K.); karayann@auth.gr (G.K.); 2 Laboratory of Pathology, Lyon-Sud Hospital Center, 69495 Pierre Bénite, France; 3 Department of Dermatology/CliMA, Ed. Herriot Hospital (Pav. R), 5 place d’Arsonval, 69437 cedex 03, Lyon, France

**Keywords:** TRPS1, immunohistochemistry, metastatic skin tumors

## Abstract

*TRPS1* (Tricho-rhino-phalangeal syndrome 1) is a GATA transcriptional activator gene encoding for a protein used as a sensitive immunohistochemical marker of breast carcinomas. In dermatopathology, TRPS1 is used as a marker of mammary and extramammary Paget’s disease and is also expressed by a variety of primary cutaneous tumors, mostly of adnexal origin. So far, very limited data exist on the expression of TRPS1 in metastatic skin tumors. We studied the immunohistochemical expression of TRPS1 in 72 cutaneous metastatic tumors from the breast (n: 19) and other origins (n: 53) in order to assess its diagnostic usefulness. The intensity of TRPS1 immunostaining was expressed as a histoscore: the product of the percentage of positive cells (scored semi-quantitatively 0–4) and the staining intensity (scored 0–3). In normal skin, nuclear TRPS1 expression was predominantly observed in cells of adnexal structures (pilosebaceous follicles and sweat glands). Eighteen (18/19, 94.7%) metastatic breast carcinomas showed diffuse and strong TRPS1 positivity (histoscore 12). Lower reactivity was found in some other metastases, including from the lung (11/22), the female genital tract (3/4), and the kidney (2/4), whereas most (20/22) metastases from the digestive system and peritoneum, along with a case of metastatic prostate carcinoma, were negative. These results suggest that a high histoscore for TRPS1 is in favor of the mammary origin of metastatic cutaneous carcinoma. Although TRPS1 is not absolutely specific or sensitive to a particular primary, we consider that it can be added to a panel of other markers when investigating the origin of a cutaneous metastasis, namely when this is the first manifestation of the neoplastic disease.

## 1. Introduction

Cutaneous metastatic tumors are rare, developing in 0.7% to 9% of patients with visceral malignancies [1]. Despite this relative rarity, the diagnosis of secondary involvement of the skin by a malignant tumor raises several challenges among clinicians and pathologists. From a pathological point of view, the differential diagnosis between primary and metastatic cutaneous neoplasms is difficult, and often impossible. In the second scenario, the identification of the primary origin of the neoplasm can also be challenging, especially in the case of an unknown primary, but is obviously very important for the optimal management of the patient [2,3]. Immunohistochemical studies are a very important ancillary technique for helping the pathologist to reach a precise diagnosis, based on the expression of specific antigenic markers by tumor cells. *TRPS1* (Tricho-rhino-phalangeal syndrome 1), also known as the “Transcriptional Repressor GATA binding 1” gene, is a GATA transcriptional activator [4] that has recently emerged as a useful immunohistochemical marker. It was first described in the context of Tricho-rhino-phalangeal syndrome (TRPS, OMIM 190350), a genetic malformative disease characterized by craniofacial and skeletal abnormalities [5]. *TRPS1* controls the proliferation and differentiation of normal mammary epithelial cells. Although it was initially reported as a highly sensitive and specific marker of epithelial and mesenchymal neoplasms of the breast [6,7], more recent data have shown that TRPS1 is expressed by various other non-breast neoplasms, including benign and malignant cutaneous ones [8,9,10]. However, very limited data exist so far regarding the expression of TRPS1 in metastatic cutaneous tumors. The aim of this study was to investigate the expression of TRPS1 in metastatic cutaneous malignances, in order namely to assess the value of this marker in diagnosing the origin of the corresponding primaries.

## 2. Materials and Methods

This study was a retrospective analysis of archival tissues. We retrieved cases of cutaneous metastatic carcinomas from the archives of the Pathology Department, AHEPA University Hospital, School of Medicine, Aristotle University of Thessaloniki (Greece), and the Pathology Laboratory of the Lyon-Sud University Hospital, Hospices Civils de Lyon (France). The cases were randomly selected using the electronic registry of each department. They included biopsy or excision specimens of metastases of known primary origin for which sufficient formalin-fixed, paraffin-embedded archival material to perform an additional immunohistochemical study was available. The diagnoses had been made by examination of hematoxylin-eosin-stained sections and appropriate immunostainings and after integration of clinical data retrieved from the patient’s medical files concerning any known history of malignancy. Routinely stained sections from each case were reviewed, and the most representative paraffin blocks were selected.

The immunohistochemical study (IHC) was performed in an automated Ventana machine on 3-micron-thick tissue sections cut from the paraffin blocks. Briefly, the sections were deparaffinized and dehydrated. Ethylene-diamine tetra-acetic acid (EDTA) was used for antigen retrieval for 64 min. The anti-TRPS1 antibody (clone ZR382, Zeta Corporation, dilution 1/100) was incubated for 32 min. The antigen–antibody complex was visualized using diaminobenzidine (DAB) as the chromogen. The slides were counterstained with Mayer’s hematoxylin for 10 min, washed in water, dehydrated, and mounted. For each case, the percentage of positive tumor cells was assessed and scored semi-quantitatively (0: negative, 1: <25% positive cells, 2: 26–49% positive cells, 3: 50–74% positive cells, 4: >75% positive cells). The staining intensity was also evaluated semi-quantitatively (0: negative, 1: mild, 2: moderate, 3: strong), considering as 3 the highest intensity observed in the most intensely labeled skin structures present in each slide, i.e., eccrine sweat glands and papillary mesenchymal cells around the hair bulb. A histoscore was calculated for each case by multiplying the two scores (percentage of positive cells and staining intensity, ranging from 0 to 12). Adnexal (pilosebaceous or sweat gland) structures served as internal positive controls in each section.

## 3. Results

### 3.1. Cases Studied

A total of 72 biopsy specimens with metastatic skin carcinomas, obtained from 40 men (55.6%) and 32 women (44.4%) aged 23–94 (mean 65.6) years, were studied (Table 1). Nineteen metastases were of mammary origin (six from invasive lobular carcinomas/ILC and 13 from invasive breast carcinomas of no special type/IBC, NST). Fifty-three metastatic cases were of different (non-breast) origin, including: 22 from the lung, 21 from the digestive system, five and four from the male and female genito-urinary tracts, respectively, and one mesothelioma derived from the peritoneum. The 22 lung tumors included 16 adenocarcinomas, two non-small-cell carcinomas, one squamous cell carcinoma (SCC), and three neuroendocrine neoplasms. The tumors of the digestive system included 12 colorectal adenocarcinomas, three adenocarcinomas of the stomach, two cholangiocarcinomas of the extrahepatic bile ducts, and one case each of SCC of the tongue, pancreatic adenocarcinoma, appendiceal adenocarcinoma, and intestinal neuroendocrine carcinoma. The 10 remaining cases included four clear cell renal carcinomas, two uterine cervix carcinomas, and one case each of prostatic adenocarcinoma, high-grade serous ovarian carcinoma, undifferentiated carcinoma (of the uterus or the ovary), and mesothelioma.

### 3.2. Immunohistochemical Expression of TRPS1

#### 3.2.1. Normal Skin

**Figure 1:**
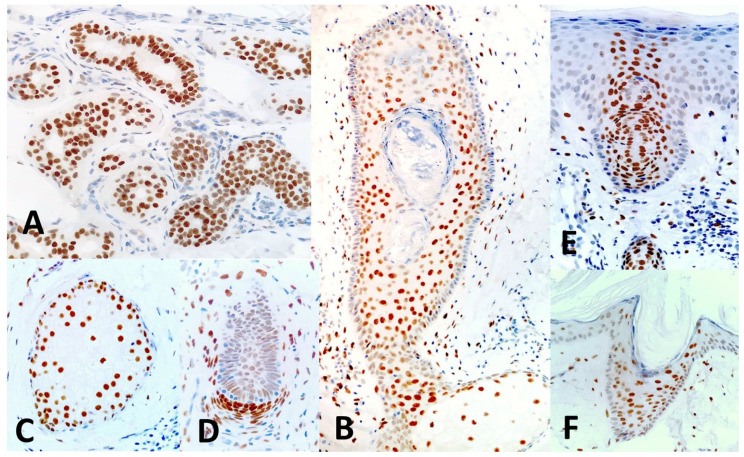
Nuclear TRPS1 expression in normal skin. TRPS1 is strongly expressed in a sweat gland coil, consisting of a secretory and an excretory segment (**A**). TRPS1 is expressed by cells of the hair follicle sheath (**B**), the sebaceous gland (**C**), and fibroblasts of the hair bulb (**D**). TRPS1 expression allows us to visualize the intraepidermal parts of the sweat ducts/acrosyringia (**E**) and the hair follicles (**F**). Immunoperoxidase revealed with diaminobenzidine, counterstaining with Mayer’s hematoxylin.

**Figure 2:**
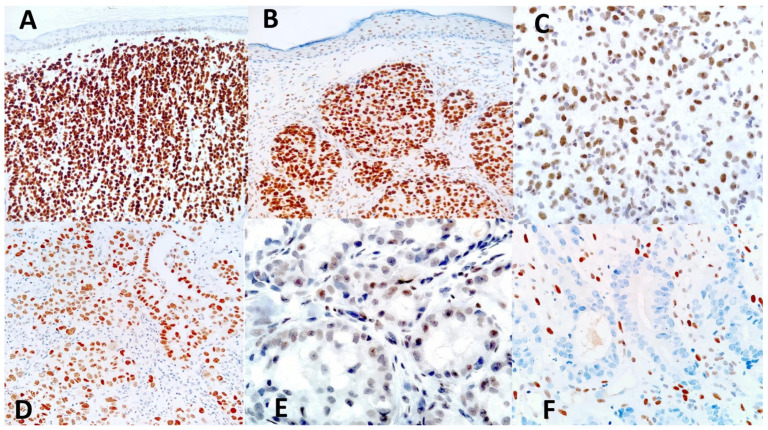
Expression of TRPS1 by metastatic skin tumors. Diffuse and strong TRPS1 expression (histoscore 12) in a metastatic breast carcinoma (**A**) and a pulmonary squamous cell carcinoma (**B**). Note the weak TRPS1 expression by epidermal keratinocytes. Variable, weaker TRPS1 expression is seen in cases of metastases from renal cell carcinoma ((**C**), histoscore 8), ovarian carcinoma ((**D**), histoscore 8), and lung carcinoma ((**E**), histoscore 3). (**F**): TRPS1-negative colon adenocarcinoma (histoscore 0). Immunoperoxidase revealed with diaminobenzidine, counterstaining with Mayer’s hematoxylin.

In normal skin adjacent to the tumors studied, nuclear expression of TRPS1 was mainly observed in the cells of adnexal structures of the epidermis, i.e., pilosebaceous follicles and sweat glands, and served as internal positive controls. Strong TRPS1 expression was found in cells of both the secretory and the excretory parts of eccrine sweat glands (Figure 1A) and in cells of the hair follicle, including the fibroblasts of the dermal follicle papilla, which showed strong expression (Figure 1B,D). TRPS1 was also expressed within the sebaceous glands, more intensely by the less differentiated peripheral sebocytes (Figure 1C). Occasionally, TRPS1 was expressed, although more faintly, by some interstitial dermal cells and by epidermal keratinocytes (Figure 2B). TRPS1 expression allowed us to visualize the intraepidermal parts of the sweat ducts (acrosyringia—Figure 1E) and of the hair follicles (acrotrichia—Figure 1F).

#### 3.2.2. Cutaneous Metastatic Tumors (Table 1)

In metastatic tumors, TRPS1 expression varied significantly among the cases studied, both in terms of percentage of labeled cells and of staining intensity. In detail, all but one (18/19, 94.7%) of the metastatic breast carcinomas showed diffuse and strong TRPS1 positivity (histoscore 12), regardless of histological subtype (Figure 2A). The TRPS1-negative case was from a female invasive breast carcinoma of no special type (grade 3 according to the Nottingham histologic score). Half of the metastatic lung carcinoma cases showed some degree of TRPS1 expression, with the case of SCC showing diffuse and strong positivity (histoscore 12) (Figure 2B,E). The remaining positive metastatic lung carcinoma cases had histoscores ranging from 2 to 8. The majority (90.9%) of metastatic carcinomas of the digestive system (from gastric and colorectal adenocarcinomas, adenocarcinoma of the appendix, SCC of the tongue, and cholangiocarcinomas) were TRPS1-negative (Figure 2F). The remaining two (positive) cases included a case of intestinal neuroendocrine neoplasm with intermediate TRPS1 positivity (histoscore 6) and one case of pancreatic duct origin with weak TRPS1 expression (histoscore 1). Concerning the urogenital malignancies, 2/4 (50%) of renal clear cell carcinomas showed intermediate TRPS1 expression (histoscore 8–Figure 2C), whereas the two other cases were negative, as was the case of prostatic adenocarcinoma. In tumors of the female genital tract, strong positivity (histoscore 12) was observed in one case of cervical carcinoma, whereas the second case was negative. Both the serous carcinomas of the ovary and the undifferentiated carcinoma of ovary/uterus were TRPS1-positive (histoscore 8—Figure 2D). One case morphologically and immunohistochemically consistent with metastatic mesothelioma showed weak and patchy (<10% of neoplastic cells) TRPS1 positivity (histoscore 1).

**Table 1:** TRPS1 immunoreactivity in cutaneous metastatic tumors.

Case n°	Age	Gender	Primary Origin	Histological Type	TRPS1-Pos (% Tumor Cells)	TRPS1-Pos (Intensity, 0–3)	Histoscore
1	70	F	breast	ILC	4	3	12
2	64	M	breast	IBC-NST	4	3	12
3	68	F	breast	IBC-NST	4	3	12
4	80	F	breast	IBC-NST	4	3	12
5	74	F	breast	ILC	4	3	12
6	90	F	breast	IBC-NST	4	3	12
7	65	F	breast	IBC-NST	4	3	12
8	37	F	breast	IBC-NST	4	3	12
9	89	F	breast	ILC	4	3	12
10	73	F	breast	IBC-NST	4	3	12
11	38	F	breast	IBC-NST	4	3	12
12	70	F	breast	IBC-NST	4	3	12
13	93	F	breast	IBC-NST	4	3	12
14	76	F	breast	ILC	4	3	12
15	66	F	breast	IBC-NST	4	3	12
16	89	F	breast	IBC-NST	4	3	12
17	67	F	breast	ILC	4	3	12
18	59	F	breast	IBC-NST	0	0	0
19	83	F	breast	ILC	4	3	12
20	68	M	lung	NET G1	0	0	0
21	94	M	lung	ADC	0	0	0
22	59	M	lung	ADC	0	0	0
23	72	M	lung	NEC	0	0	0
24	54	M	lung	SCC	4	3	12
25	80	M	lung	ADC	4	2	8
26	69	M	lung	ADC	4	2	8
27	73	M	lung	ADC	0	0	0
28	71	M	lung	ADC	2	1	2
29	80	M	lung	ADC	3	2	6
30	54	F	lung	NSCLC	0	0	0
31	74	M	lung	ADC	0	0	0
32	53	M	lung	ADC	3	1	3
33	68	M	lung	ADC	4	2	8
34	66	M	lung	SCLC	4	2	8
35	59	F	lung	ADC	4	2	8
36	73	M	lung	ADC	2	1	2
37	74	F	lung	ADC	0	0	0
38	84	F	lung	ADC	0	0	0
39	63	M	lung	ADC	0	0	0
40	64	M	lung	ADC	4	1	4
41	65	M	lung	NSCLC	0	0	0
42	49	M	peritoneum	MST	1	1	1
43	53	M	tongue	SCC	0	0	0
44	42	F	rectum	ADC	0	0	0
45	70	M	colon	ADC	0	0	0
46	70	M	colon	ADC	0	0	0
47	69	M	sigmoid	ADC	0	0	0
48	70	M	rectum	ADC	0	0	0
49	58	M	colon	ADC	0	0	0
50	23	M	rectum	ADC	0	0	0
51	49	M	colon	ADC	0	0	0
52	73	M	colon	ADC	0	0	0
53	72	M	colon	ADC	0	0	0
54	66	M	colon	ADC, NOS	0	0	0
55	NA	M	colon	ADC, NOS	0	0	0
56	59	F	stomach	PCC, NOS	0	0	0
57	47	M	stomach	PCC, NOS	0	0	0
58	63	M	stomach	ADC, tubular	0	0	0
59	62	M	intestine	NEC	3	2	6
60	48	M	appendix	ADC	0	0	0
61	87	M	extrahepatic bile ducts	CHC	0	0	0
62	67	F	extrahepatic bile ducts	CHC	0	0	0
63	45	M	pancreas	ADC, ductal	1	1	1
64	NA	M	kidney	CCRCC	4	2	8
65	58	F	kidney	CCRCC	4	2	8
66	69	F	kidney	CCRCC	0	0	0
67	NA	M	kidney	CCRCC	0	0	0
68	73	M	prostate	ADC	0	0	0
69	47	F	cervix	ADC	4	3	12
70	47	F	cervix	ADC, mucinous	0	0	0
71	60	F	ovary or uterus	UNDC	4	2	8
72	64	F	ovary	HGSC	4	2	8

Abbreviations: ADC: adenocarcinoma, CHC: cholangiocarcinoma, CCRCC: clear cell renal cell carcinoma, HGSC: high grade serous carcinoma, IBC-NST: invasive breast carcinoma-no special type, ILC: invasive lobular carcinoma, MST: mesothelioma, NEC: neuroendocrine carcinoma, NET G1: neuroendocrine tumor, grade 1, NOS: not otherwise specified, NSCLC: non-small-cell carcinoma, PCC: poorly cohesive carcinoma, SCC: squamous cell carcinoma, SCLC: small-cell lung carcinoma, UNDC: undifferentiated carcinoma.

## 4. Discussion

The microscopic diagnosis of cutaneous metastases is often fraught with difficulty. Indeed, although these tumors can often be broadly categorized into adenocarcinomas or squamous cell carcinomas, they seldom display specific histological features that allow recognition of the primary site of origin. This situation is especially challenging when the metastasis is the first manifestation of an underlying, hitherto unknown malignancy [2,11]. In this scenario, the search for specific and sensitive immunohistochemical markers provides useful, often invaluable, diagnostic information to the pathologist. In the setting of metastatic tumors, knowledge of the expression of a given marker by the primary tumors is certainly of paramount importance. TRPS1 is an emerging immunohistochemical marker that has been used as a sensitive and specific marker of breast carcinoma [6,7] and of mammary and extramammary [12,13] Paget’s disease. More recent studies have shown that TRPS1 is also expressed, although less frequently, by other, non-breast malignancies [6,14].

The expression of TRPS1 in normal and diseased skin has been previously reported [8,9,10,15,16,17]. Our results concerning normal skin align with the results of these studies, showing TRPS1 expression predominantly in cells of adnexal structures of the epidermis, i.e., pilosebaceous follicles and sweat glands. Of note is the fact that epidermal keratinocytes occasionally express TRPS1, and this could be related to the degree of ultraviolet-induced DNA damage [16]. Regarding tumoral pathology, the expression of TRPS1 has been studied in primary tumors of the skin [8,9,10,15,16,17]; however, very limited data exist on cutaneous metastatic tumors, as only one study has investigated a small number of such cases [8]. In the present study, we aimed to assess the usefulness of TRPS1 in the diagnosis of cutaneous metastatic tumors by studying a larger patient cohort. We found that the vast majority (18/19, i.e., 94.7%) of cutaneous metastases of mammary origin (both from ILC and IBC-NST primaries) showed strong TRPS1 expression (histoscore 12). This result is in keeping with results in the literature showing very frequent and strong TRPS1 expression by primary breast tumors [6,7,18,19,20] and with the study by Taniguchi et al. [8], which found all eight cases to be TRPS1-positive. Remarkably, in our study, we found one case of TRPS1-negative metastatic breast carcinoma. This is consistent with the fact that 9.6% of primary breast carcinomas are TRPS1-negative [7], and indicates that, even though strong TRPS1 positivity favors the mammary origin of a metastatic tumor, negativity of TRPS1 cannot completely rule out this origin of a cutaneous metastasis.

Regarding the remaining metastatic tumors of non-breast origin, we found TRPS1 expression in over one-third of all cases, including metastases from the lung, pancreas, kidney, and gynecologic tract. This result is consistent with the results of previous studies showing expression of TRPS1 by the corresponding primary malignancies, including those of the endometrium and ovary [21,22], salivary glands [23,24], and the gastrointestinal tract [25]. Of note, however, is the fact that, in contrast to the mammary metastases, the metastases of non-mammary origin expressed TRPS1 in a weaker and more focal pattern, with the exception of a case of small-cell lung carcinoma that presented diffuse and strong TRPS1 positivity. Remarkably, we found all three cases of gastric adenocarcinoma to be TRPS1-negative. Considering that gastric poorly cohesive or Lauren diffuse-type carcinomas often share common morphological and immunohistochemical features with ILC [26,27], we believe that TRPS1 may serve as an interesting marker in the differential diagnosis between these two tumor types.

The rather high percentage of TRPS1-positive metastatic non-mammary tumors we found (36.5%) is at some variance with a previous study [8], that found only 1/11 of cutaneous metastasis of a lung carcinoma to express TRPS1 weakly, and with the results of Ai et al. [6], who found a rather low percentage of visceral (non-mammary) tumors to be TRPS1-positive. These discrepancies could be due to several reasons: (a) the study of Ai et al. [6] used tissue microarrays and may therefore have missed the TRPS1-positive parts in tumors with focal TRPS1 positivity; (b) differences in the immunohistochemical protocol used (different antibody); (c) finally, the possibility exists that metastatic cells show an upregulated TRPS1 expression compared with the primary tumors of origin, as some studies have suggested a possible prognostic significance of TRPS1 in specific tumors [4,21,22]. This issue could be solved by comparing metastases to the skin (or other site) with their corresponding primaries.

Another important diagnostic problem when facing a malignant skin tumor is the recognition of its primary (cutaneous) vs. secondary (metastatic) nature. Several markers have been investigated to this end (such as hormone receptors, p63, p40, several keratin polypeptides), but it seems that none is absolutely specific so as to reliably distinguish metastatic tumors from primary ones [28,29], especially in the case of adenocarcinomas, which can be metastatic (namely from the breast) or originate from the sweat gland apparatus of the skin. Data from the literature have shown that both benign and malignant adnexal skin tumors express TRPS1 to various degrees [8,9,10,15,16,17]. However, normal apocrine sweat glands [15,16] and apocrine carcinomas [16] were reported to be TRPS1-negative, suggesting that TRPS1-positivity in a cutaneous adenocarcinoma could favor its metastatic, rather than primary, origin. Of note, however, is the fact that one recently-published case of apocrine carcinoma carrying a RARA::NPEPPS fusion was found to be TRPS1-positive [30]. A larger number of apocrine carcinomas need therefore to be studied in order to definitively assess the usefulness of TRPS1 in the differential diagnosis between primary and metastatic adenocarcinomas of the skin.

Our study has some limitations, including namely the relatively small size of the cohort studied and the low representation of some TRPS1-positive tumor types, such as metastases from SCC and from mesenchymal tumors such as leiomyosarcomas. Furthermore, due to the unavailability of the primary tumors, we could not compare the degree of TRPS1 expression in the metastases with their corresponding primaries, an interesting topic that certainly merits investigation.

## 5. Conclusions

We present here the largest cohort of metastatic carcinomas to the skin studied for the expression of TRPS1. Our findings suggest that strong expression of TRPS1 favors a mammary origin of a cutaneous metastasis, although it cannot exclude a different primary origin, namely from the lung, the kidney, or the female genital tract. Conversely, negativity for TRPS1 cannot formally exclude the mammary origin of a cutaneous metastasis. A comprehensive clinicopathologic correlation, including imaging studies, is certainly important in this setting. With these limitations in mind, we consider that TRPS1 can be included in a panel of other immunohistochemical markers for the diagnosis of cutaneous metastatic tumors, in the context of adequate clinical information. Further studies, comparing namely the expression of metastatic tumors with their primaries of origin, will shed more light on the significance of TRPS1 expression in metastatic skin tumors, namely its value as a diagnostic and/or prognostic marker.

## Data Availability

The original contributions presented in the study are included in the article. Further inquiries can be directed to the corresponding author.
